# Estimating generation time of SARS-CoV-2 variants in Italy from the daily incidence rate

**DOI:** 10.1038/s41598-023-38327-y

**Published:** 2023-07-17

**Authors:** Eugenio Lippiello, Giuseppe Petrillo, Silvio Baccari, Lucilla de Arcangelis

**Affiliations:** 1 Department of Mathematics and Physics, University of Campania “L. Vanvitelli”, Viale Lincoln, 5, 81100 Caserta, Italy; 2 The Institute of Statistical Mathematics, Research Organization of Information and Systems, Tokyo, Japan

**Keywords:** Complex networks, Nonlinear phenomena, Phase transitions and critical phenomena

## Abstract

The identification of the transmission parameters of a virus is fundamental to identify the optimal public health strategy. These parameters can present significant changes over time caused by genetic mutations or viral recombination, making their continuous monitoring fundamental. Here we present a method, suitable for this task, which uses as unique information the daily number of reported cases. The method is based on a time since infection model where transmission parameters are obtained by means of an efficient maximization procedure of the likelihood. Applying the method to SARS-CoV-2 data in Italy, we find an average generation time ${\overline{z}}=3.2 \pm 0.8$ days, during the temporal window when the majority of infections can be attributed to the Omicron variants. At the same time we find a significantly larger value ${\overline{z}}=6.2\pm 1.1$ days, in the temporal window when spreading was dominated by the Delta variant. We are also able to show that the presence of the Omicron variant, characterized by a shorter ${{\overline{z}}}$, was already detectable in the first weeks of December 2021, in full agreement with results provided by sequences of SARS-CoV-2 genomes reported in national databases. Our results therefore show that the novel approach can indicate the existence of virus variants, resulting particularly useful in situations when information about genomic sequencing is not yet available. At the same time, we find that the standard deviation of the generation time does not significantly change among variants.

## Introduction

SARS-CoV-2, as other viruses, are continuously evolving because of genetic mutations or viral recombination. These changes can strongly affect transmission parameters^1^ inducing important differences in the virus spreading. In particular a reduction of the generation time *z*, i.e. the time difference between the dates of infection of successive cases in a transmission chain, leads to an increased epidemic growth rate, even for unaltered reproduction number $R_0$. Furthermore, an accurate estimate of the mean value of the generation time ${\overline{z}}$ is fundamental to establish the optimal duration of the quarantine period.

Elegant methods based on log-likelihood maximization have been recently developed^2–5^ to obtain the average value ${\overline{z}}$ of the generation time. However, very often ${\overline{z}}$ is identified with ${\overline{s}}$, defined as the mean value of the serial interval^4^, which is the difference in timing of symptom onset in a pair of a primary and its secondary case. The measurement of ${\overline{s}}$, indeed, differently from the measurement of ${\overline{z}}$, can be directly obtained from the reconstruction of the contact network. This information, combined with the results provided by genomic sequencing, provides an estimate of the mean value of serial intervals of each specific variant^6^. Nevertheless, it is important to remark^7–10^ that the value of ${{\overline{s}}}$, obtained from contact tracing can be significantly different than the “intrinsic” value of ${{\overline{z}}}$. This occurs, in particular, when the structure of the contact network fastly changes in time, as for instance in presence of non-pharmaceutical interventions. Here “intrinsic” refers to the quantity measured in the ideal case of a fully susceptible, homogeneously mixed population^7^, and therefore independent of the specific conditions of the epidemiological setting from which it is inferred.

In this study we will show that the intrinsic value of ${\overline{z}}$ can be obtained by means of a completely data driven procedure. The main observation is that, if the value of ${\overline{z}}$ affects the future evolution of the number of infected cases, its value could be potentially extracted from the previous evolution of the virus spreading. More precisely we use the method recently developed by us^5^ to extract ${\overline{z}}$ directly from the daily series of incidence rate *I*(*t*), i.e. the number of infected individuals at the calendar time *t*. The method is based on the non-trivial dependence on ${\overline{z}}$ of the Log-Likelihood function *LL*, which measures the overlap between the measured *I*(*t*) and the expected one, according to a time since infection model^11,12^. We show that when two variants with differences in ${\overline{z}}$ are simultaneously present in the sample, $LL({\overline{z}})$ presents two distinct peaks in correspondence to the mean value of *z* of the two dominant variants. Furthermore the ratio between the two peak heights also provide information about the relative incidence of the two variants in virus spreading.

We perform this study using the incidence rate *I*(*t*) for SARS-CoV-2 in Italy where three Variants of Concern (VOC) have dominated in three different temporal windows, as highlighted in Table  1 using the information present in the Bulletin (No. 3 to 21) of Istituto superiore di Sanitá (www.epicentro.iss.it) (see also Fig. 3report-n.21).

**Table 1:** Percentage of diffusion of major variants in Italy from 28 December, 2020, to June 27, 2022, based on weekly sampling and data provided by the I-Co-Gen platform software.

Starting date ending date	12/28/20 6/6/21 (%)	6/5/21 7/19/21 (%)	7/17/21 8/30/21 (%)	8/28/21 10/11/21 (%)	10/9/21 11/22/21 (%)	11/27/21 1/10/22 (%)	2/5/22 3/21/22 (%)	4/9/22 5/23/22 (%)	5/14/22 6/27/22 (%)
Alpha	74.9	35.9	2.3	0	0.3	1.2	0	0	0
Beta	0.8	0.3	0	0	0	0	0	0	0
Gamma	6.3	7.2	0.4	0	0	0.1	0	0	0
Eta	1.2	0.6	0.1	0	0	0	0	0	0
Kappa	0.3	1.3	1.2	1.5	0	0	0	0	0
Delta	–	46.0	88.1	90.8	91.4	66.7	0.7	0.1	0
Omicron-BA1	–	–	–	–	–	15.5	63.7	4.4	5.5
Omicron-BA2	–	–	–	–	–	–	27.3	82.0	56.0

Data are extracted from the reports titled “Prevalenza e distribuzione delle varianti di SARS-CoV-2 di interesse per la sanità pubblica in Italia”, from Report No. 3, dated June 25, 2021 up to Report No. 21, dated July 1, 2022, available at the official website of the Italian National Institute of Health (ISS) www.epicentro.iss.it. Different rows correspond to different SARS-CoV-2 vartiants, different columns correspond to different temporal windows.

Several studies^6,13–15^ have measured, in different geographic regions, a value ${{\overline{s}}}$ of the Omicron variant significantly shorter than the value measured for previous variants Alpha and Delta. Because of this observation, many countries have applied a reduction of the duration of the quarantine period (www.ecdc.europa.eu). This is in agreement with the evaluation of the intrinsic value of ${{\overline{z}}}$ using nucleotide sequences of SARS-CoV-2 viruses sampled in Denmark^16^, leading to a value of ${{\overline{z}}}$ for the Omicron variant about 0.5–0.6 times smaller than the one measured for the Delta variant. Conversely, a study of infections among household members in Reggio Emilia (Italy) lead to an intrinsic value of ${{\overline{z}}}$ which is about 6 days, with no significant difference among the three variants, Alpha, Delta e Omicron^10^. Our data appear more consistent with the result of^16^ since we find a value ${{\overline{z}}}=3.2 \pm 0.8$ days to the Omicron variant respect to the value $\overline{z}=6.5 \pm 1$ days associated to the Delta one.

## The method

In this section, we overview the method considered in this study, details can be found in^5^.

The starting point is the renewal equation^11,12,17^ providing the expected value of daily infected people on the *m*-th day, *E*[*I*(*m*)], in terms of the past daily incidence

1 $$\begin{aligned} E[I(m)]=\sum _{j=0}^{m-1}R_c(j) w(m-j)I(j) +\mu (m), \end{aligned}$$

where $R_c(m)$ is the case reproduction number, representing the total number of infections induced on average by an individual infected on the *m*-th day, *w*(*j*) is the distribution of generation times, representing the percentage of infections induced at a time distance *j* from the infection and, finally, $\mu (m)$ is the daily number of imported cases during the *m*-th day, i.e. infectors coming from outside the considered region. We assume that *w*(*j*) is a Gamma distribution, $w(j)=\left( \tau ^{-a}/\Gamma (a) j^{a-1}\right) \exp (-j/\tau )$, which depends on two parameters, $a\ge 1$ and $\tau >0$, and where $\Gamma (a)$ is the Gamma function. The Gamma distribution is fully characterized by its average value ${{\overline{z}}}$ and by its standard deviation $\sigma$, which are both functions of *a* and $\tau$, ${{\overline{z}}}=a \tau$ and $\sigma =\sqrt{a} \tau$. In Supplementary Information (SI) we show that similar results are found for a Weibull or a log-normal distribution (Figs. Suppl.3, 4). In SI, we also show (Fig. Suppl. 5) that $\sigma$ weakly affects the reprouction number $R_c(m)$ but, as deeply discussed in^5^, it remains an important parameter in defining the appropriate length of quarantine.

An analytical expression for the log-likelihood *LL* of the time series $\{I(m)\}_{m=1,\ldots ,N}$, for assigned sequences $\{R_c(m)\}_{m=1,\ldots ,N}$, $\{\mu (m)\}_{m=1,\ldots ,N}$, and for given values of ${{\overline{z}}}$ and $\sigma$ has been obtained^5^ under the hypothesis that the number of individuals infected on the *m*-th day is Poisson distributed. For fixed ${{\overline{z}}}$ and $\sigma$, the best series $\{R_c(m)\}_{m=1,\ldots ,N}$ and $\{\mu (m)\}_{m=1,\ldots ,N}$ which maximize *LL* are finally obtained by generalizing the Markov-chain-Monte-Carlo method introduced to find the optimal parameters in epidemic models for seismic occurrence^18,19^.

In the following, we define $LL^{best}({{\overline{z}}},\sigma )$, the value of *LL* in correspondence to the best series $\{R_c(m)\}_{m=1,\ldots ,N}$ and $\{\mu (m)\}_{m=1,\ldots ,N}$ and we explore its dependence on the parameters ${{\overline{z}}}$ and $\sigma$. The identification of $LL^{best}({{\overline{z}}},\sigma )$ allows us to obtain also an accurate estimate of $\sigma$, which represents a measure of the duration of the infectious period and which is difficult to be obtained by contact tracing^2,3^. The numerical code is available for open access at github-algorithm. The pipeline can be found in Fig. Suppl. 1.

## Results

**Figure 1:**
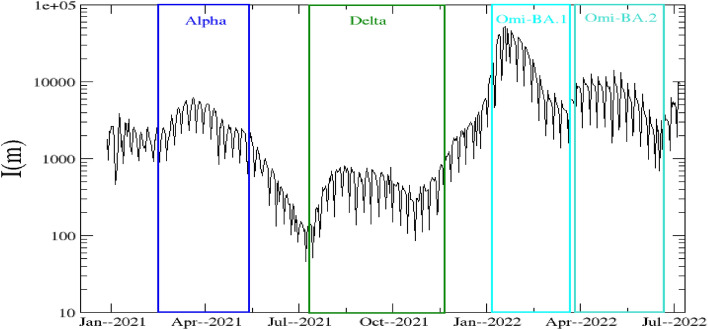
The daily incidence *I*(*m*) of SARS-CoV-2 in Lombardy from January 2021 up to June 2022. Colored boxes identify the four different temporal windows Alpha, Delta, Omicron-BA.1 and Omicron-BA.2 obtained from Table 1.

We consider data provided by the Department of Protezione Civile in Italy. More precisely we mainly consider data for the region Lombardy where the first outbreak of SARS-CoV-2 has been documented in Europe and which is characterized by a widespread diffusion of the disease since March 2020. In Fig. 1, we plot the daily incidence from January 2021. In the figure, we highlight the three main temporal windows which, according to the results of Table 1, are mostly characterized by the spreading of a specific variant. We have also identified two sub-windows where the spreading is mainly controlled by two different lineages of Omicron BA.1 and BA.2, respectively. It is evident that each temporal window corresponds to a different wave of Covid spreading with a distinct peak in *I*(*m*).

**Figure 2:**
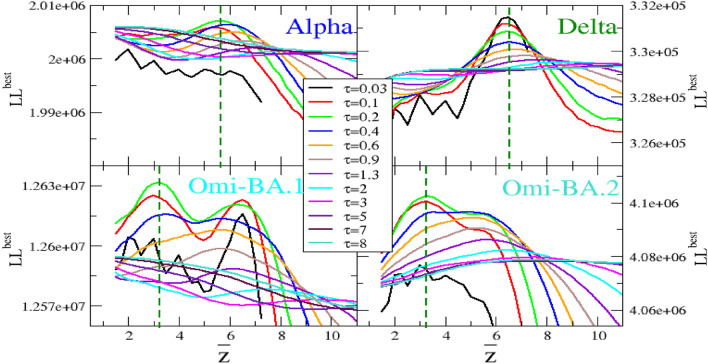
The log-likelihood $LL^{best}({{\overline{z}}},\sigma )$, evaluated for the temporal profile of $R_c(m)$ which maximizes the likelihood for the daily incidence of SARS-CoV-2 in Lombardy, is plotted as a function of ${{\overline{z}}}=a \tau$. The four different panels correspond to the four temporal windows highlighted in Fig. 1. Different curves, in each panel, correspond to different values of $\tau$, which implies a different $\sigma =a \sqrt{\tau }$. The dashed green vertical line identifies the value of ${{\overline{z}}}$ which provides the maximum value of the log-likelihood, in each panel.

We separately apply the procedure outlined in the previous section, restricting to data within each of the 4 temporal windows, which are classified as Alpha, Delta, Omicron-BA.1 and Omicron-BA.2. The evolution of the reproduction number $R_c(m)$ and of the daily number of imported case $\mu (m)$ is reported in Fig. Suppl. 2.

The values of $LL^{best}({{\overline{z}}},\sigma )$, for different choices of ${{\overline{z}}}$ and $\sigma$, for each of the four temporal windows are plotted in a separate panel of Fig. 2. More precisely, we plot $LL^{best}({{\overline{z}}},\sigma )$ versus ${{\overline{z}}}$ and different values of the parameter $\tau$ of the Gamma distribution. Results clearly show that during the Alpha window, $LL^{best}({{\overline{z}}},\sigma )$ presents a clear maximum for ${{\overline{z}}}=5.7$ days when $\tau =0.2$ days, leading to an estimate ${{\overline{z}}}=5.7$ days and $\sigma =1.1$ days, consistently with previous findings both in terms of serial interval and intrinsic generation time. During the Delta period the peak is even more pronounced at ${{\overline{z}}}=6.5$ days for $\tau =0.03$ days, consistently with previous results. Interestingly, during the Omicron-BA.1 window the maximum of $LL^{best}$ at ${{\overline{z}}}=6.5$, observed during the Delta window, is still present but is subleading, and the most relevant peak is present at a significantly smaller values ${{\overline{z}}}=3.2$ days for $\tau =0.2$ days. During the Omicron-BA.2 window the peak at ${{\overline{z}}}=3.2$ days is the only relevant one presented by $LL^{best}$. Results of Fig. 2 clearly show a significant reduction of the generation time of the Omicron variants, with an estimated value ${{\overline{z}}} =3.2$ days which is roughly half of the value estimated during the Delta period, in agreement with results of serial intervals and of Ref.^16^. On the other hand, we do not find significant differences for the value of ${{\overline{z}}}$ between the two Omicron lineages BA.1 and BA.2. Figure 2 also gives $\sigma =0.8$ days for both Omicron lineages. This result, compared with $\sigma =1.1$ days measured during the Delta period, indicates that the standard deviation is similar for the different variants.

The analysis of Fig. 2 clearly indicates a significant reduction in the average generation time, ${\overline{z}}$, for the Omicron variants. Specifically, ${\overline{z}}$ is roughly half the estimated value observed during the Alpha and Delta time windows. This finding is consistent with the results presented in Ref.^16^, where a similar conclusion was drawn based on the analysis of the serial interval distribution. Notably, we obtained similar estimates of ${\overline{z}}$ and $\sigma$ for the other Italian regions in each of the four temporal windows (Figs. Suppl. 7–10). Additionally, we demonstrate in SI that our estimate of ${\overline{z}}$ is minimally affected by underestimates of the daily incidence rate *I*(*m*) due to unreported cases (Fig. Suppl. 6).

We remark that in the presence of two peaks of $LL^{best}$, if the range of parameters is not completely explored, it may occur that automatic procedures for log-likelihood maximization, based on Monte Carlo Markov Chains, could remain trapped in a relative maximum without reaching the global one. The result of Ref.^10^ could be affected by this problem identifying as best model parameters the ones related to the Delta peak instead of those associated to the Omicron one.

**Figure 3:**
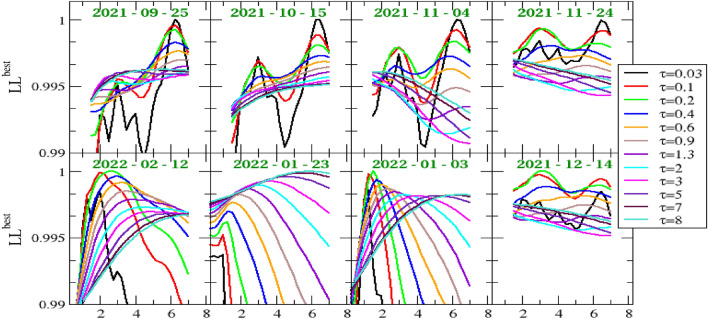
The log-likelihood $LL^{best}({{\overline{z}}},\sigma )$ is plotted as a function of ${{\overline{z}}}=a \tau$. Different panels correspond to different temporal windows of 60 days, with the initial day of each window reported on the top of each panel. The value of $LL^{best}({{\overline{z}}},\sigma )$ has ben divided by its maximum value $LL^{best}({{\overline{z}}}^{max},\sigma ^{max})$ in each temporal window, so that the maximum value of $LL^{best}$ is normalized to 1 in each panel. Panels are organized in such a way that the initial time of temporal windows increases moving from left to right in upper panels and then continuous to increase moving in the lower panel from right to left. Different curves, in each panel, correspond to different values of $\tau$, which implies a different $\sigma =a \sqrt{\tau }$.

In Fig. 3, we present the behavior of $LL^{best}(\overline{z},\sigma )$ as function of ${{\overline{z}}}$ within temporal windows of a fixed duration of 60 days, with different starting days, ranging from the first one which is fully contained within the Delta window up to the last one which is fully inside the Omicron one. Results show that in the temporal window starting on 2021-09-25 (first upper panel) only the peak at ${{\overline{z}}} \simeq 6.5$ is visible in $LL^{best}$. By shifting forward the starting time and considering a time window starting on 2021-10-15 (second upper panel) a subleading peak at ${{\overline{z}}} \simeq 3$ appears. This second peak in $LL^{best}$ therefore signals the presence of a new variant with a different ${{\overline{z}}}$ in the first weeks of December 2021. This is fully consistent with the results of Table 1 indicating that the percentage of infections caused by the Omicron variant starts to be significant in the first weeks of December 2021. Moreover, consistently with Table 1, Fig. 3 shows that by further shifting forward the starting day (upper panels form left to right) the peak at ${{\overline{z}}} \simeq 3$ becomes increasingly more relevant until it turns on the dominant one in the temporal window starting on 2021-12-14 (fourth lower panel). This is again consistent with the results of Table 1 indicating that the Omicron is the most relevant variant after the mid-December 2021. Keeping on shifting forward the starting time (lower panels from right to left) the peak at ${{\overline{z}}} \simeq 3$ becomes more visible remaining the only relevant one in $LL^{best}(\overline{z},\sigma )$ in the time window starting on 2022-02-12. We remark that no clear indication can be extracted from $LL^{best}(\overline{z},\sigma )$ in the temporal window starting on 2022-01-23. In this case indeed a clear peak is not visible and the largest value of $LL^{best}$ is obtained for the largest considered value of $\tau =8$ days, indicating that the standard deviation can be as large as 10 days and therefore does not allow us to obtain any information on ${{\overline{z}}}$. We have no clear justification for the very peculiar behavior of $LL^{best}$ in this temporal window, which corresponds to the period when *I*(*m*) is in a fast decreasing phase. It could be possible that new infections within this time window are too few to extrapolate transmission parameters from *I*(*m*).

**Figure 4:**
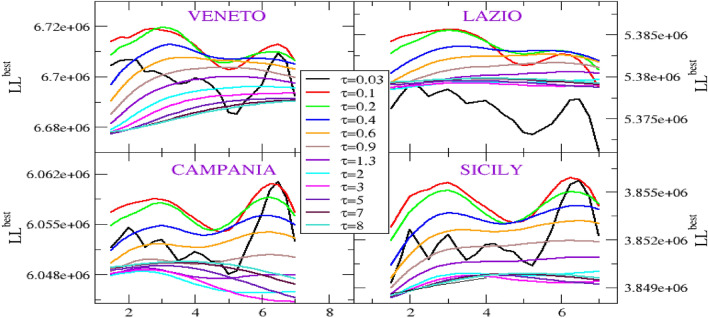
The log-likelihood $LL^{best}({{\overline{z}}},\sigma )$ is plotted as a function of ${{\overline{z}}}=a \tau$ during the Omicron-BA.1 temporal window, for four Italian regions: Veneto (upper left panel), Lazio (upper right panel), Campania (lower left panel) and Sicily (lower right panel). Different curves, in each panel, correspond to different values of $\tau$, which implies a different $\sigma =a \sqrt{\tau }$.

In Fig. 4, we consider the behavior of $LL^{best}(\overline{z},\sigma )$ in different Italian regions during the Omicron-BA1 window. Results suggest the simultaneous presence of the two variants Delta and Omicron in all the considered regions. Indeed, in all regions the two peaks at ${{\overline{z}}} \simeq 3$ and ${{\overline{z}}} \simeq 6$ are clearly visible. However, the relevance of the two peaks is different among the different regions. Indeed, in some regions like Lazio, the Omicron variant clearly appears as the dominant one during the Omicron-BA.1 window. Conversely, in Campania the contagion appears still more controlled by the Delta variant whereas in Sicily the two variants appear to contribute in a similar way to SARS-CoV-2 diffusion. In Veneto, finally, one recovers a situation very similar to the one of Lombardy (Fig. 2) with a small predominance of the Omicron variant with respect to the Delta one.

## Conclusions

We have considered an epidemic model based on a renewal equation (Eq. 1) which depends on the transmission parameters $R_c(m)$, representing the time dependent case reproduction number, and on the parameters ${\overline{z}}$ and $\sigma$, representing the mean value and the standard deviation, respectively, of the generation time distribution. We have used this model to describe the daily incidence rate of SARS-CoV-2 *I*(*m*) in Italian regions during different temporal windows. More precisely, we have obtained the value of model parameters providing the best description of experimental data by using the log-likelihood maximization procedure introduced in^5^. In particular, we have separately considered data in four different temporal windows corresponding to periods when the diffusion of SARS-CoV-2 was mostly controlled by one of the four variants (Alpha, Delta, Omicron-BA1 and Omicron-BA2). We have found that ${\overline{z}}$ during the Omicron windows was significantly smaller than, about one half of the value measured during Alpha and Delta windows, consistently with previous results about serial intervals^6,13–15^ and an estimate of ${\overline{z}}$ in Denmark^16^. By studying the behavior of the log-likelihood in different time windows, we find a clear indication of the presence of the Omicron variant in Italy since the first weeks of December 2021 with a diffusion becoming more and more relevant at later times. Our results are fully consistent with the relative diffusion of the different SARS-CoV-2 variants identified by sequencing provided by the I-Co-Gen platform software over the Italian territory. At the same time, we find that the standard deviation $\sigma$ does not differ significantly in the different time windows.

Summarizing, our study shows that the adopted procedure can be very useful to identify, in about real time, changes in the transmission parameters of a virus that can be attributed to its mutations. We remark that this result can be obtained only from the daily number of infected individuals without any further information about the identification of the correct infector–infectee pair, ignoring the timing of symptom onsets as well as other details which are necessary to reconstruct the transmission chain in traditional approaches. More importantly, our approach does not need the support of laboratory analysis for genomic sequences, which is not always available. Accordingly, the procedure adopted in this manuscript could be particularly useful in the early stage of a new pandemic, or in the early stage of a new mutation, when the genetic information on the virus is not yet complete and genomic classification is not yet available. This procedure also allows one to monitor the evolution of the standard deviation $\sigma$, which is an estimate of the duration of the infection period, an information complicated to be extracted by usual approaches based on genomic sequencing and contact tracing.

## Supplementary Information

Supplementary Information.

## Data Availability

The datasets analysed during the current study are provided by Protezione Civile for the 21 Italian regions and collected in the repository data.
