# Supplementary material for: Estimating generation time of SARS-CoV-2 variants in Italy from the daily incidence rate
Source: Sci Rep. 2023 Jul 17;13:11543. doi: 10.1038/s41598-023-38327-y (PMC10352265; doi:10.1038/s41598-023-38327-y)
Supplement: Supplementary file 1 — Supplementary Information. [file 41598_2023_38327_MOESM1_ESM.pdf]

SUPPLEMENTARY INFORMATION:Estimating generation time of SARS-CoV-2 variants in Italy from the daily incidence rate

E. Lippiello, S. Baccari, and L. de Arcangelis
Department of Mathematics and Physics, University of Campania "Luigi Vanvitelli", 81100, Caserta, Italy

G. Petrillo
The Institute of Statistical Mathematics, Research Organization of Information and Systems, Tokyo, Japan

PIPELINE OF THE ALGORITHM WORKFLOW

In Fig. Suppl.1, we present the pipeline of the numerical code used for the graphical evaluation of $\overline{z}$ and σ from the daily incidence rate. The numerical code is available in both Fortran and Python and can be accessed openly at github-algorithm

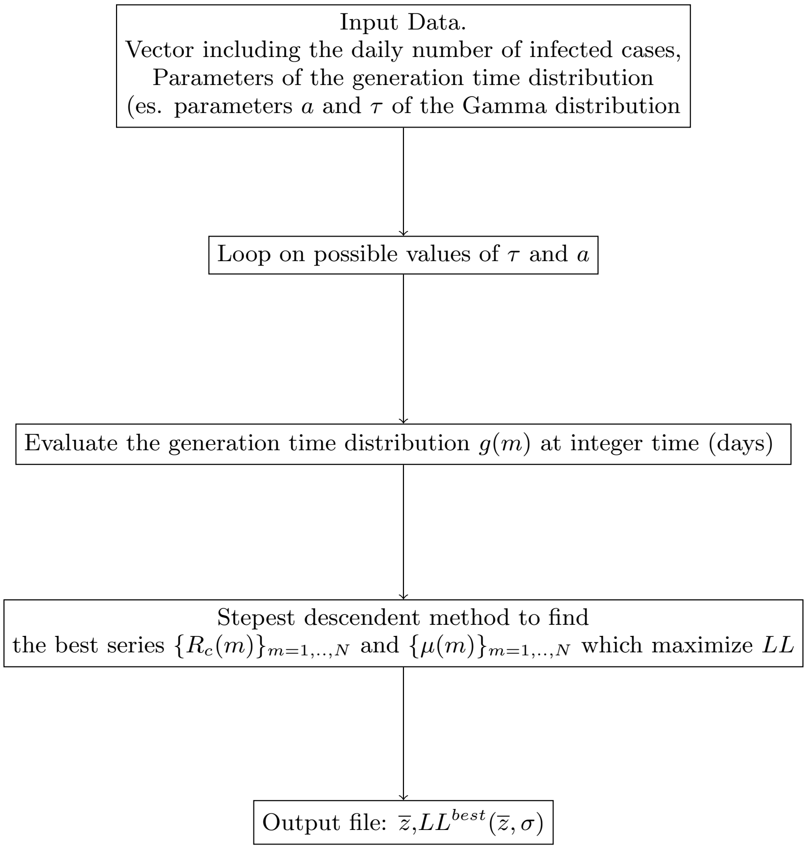

FIG. Suppl.1: Pipeline of the algorithm

TIME EVOLUTION OF $R_c(m)$ AND $\mu(m)$.

In Fig.Suppl.2 we plot the temporal variation of $R_c(m)$ and $\mu(m)$, which have been obtained for $\bar{z} = 5.8$ and $\sigma = 0.95$ corresponding to the maximum of LL during the Alpha temporal window.

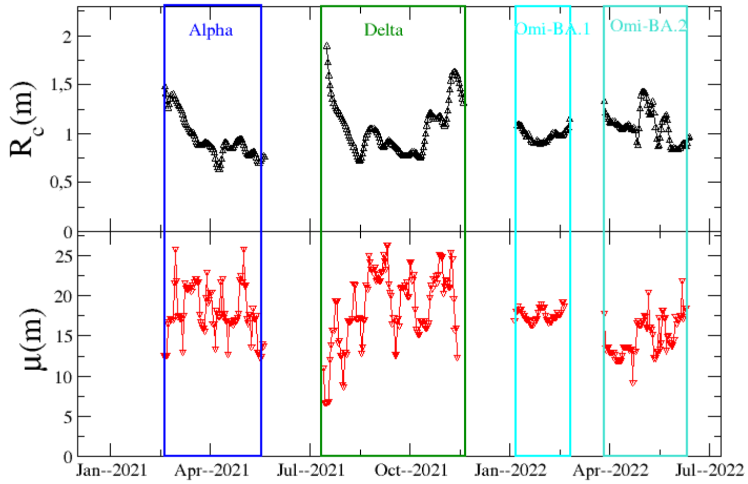

FIG. Suppl.2: (Upper panel) The weekly average of the case reproduction number $R_c(m)$ of COVID-19 for the Lombardy in the four different temporal windows Alpha, Delta, Omicron-BA1 and Omicron-BA2. (Lower panel) The weekly average of the daily number of imported cases $\mu(m)$ estimated by the log-likelihood maximization procedure.

I. INVERTING BY IMPLEMENTING A LOG-NORMAL AND A WEIBULL DISTRIBUTED $w(z)$

In Fig. 2 of the main text, we present the dependence of $LL^{best}(\overline{z},\sigma)$ on the optimal series $\{R_c\}$ and $\{\mu\}$ for different choices of the parameters a and τ in the Gamma distributed $w(z)$.

In this section, we perform the same study but assume that $w(z)$ follows either a log-normal distribution ($w(z) = \frac{k}{\sqrt{2\pi}\lambda z} e^{-\frac{(\log(z)-\mu)^2}{2\lambda^2}}$) or a Weibull distribution ($w(z) = \frac{\mu}{\lambda}\left(\frac{z}{\lambda}\right)^{\mu-1} e^{-\left(\frac{z}{\lambda}\right)^{\mu}}$).

The dependence of both distributions on two parameters, μ and λ, can be fully expressed in terms of the average value $\overline{z}$ and standard deviation σ. Specifically, for the log-normal distribution, we have $\overline{z} = e^{\mu+\lambda^2/2}$ and $\sigma = \overline{z}\left(e^{\lambda^2} - 1\right)$. For the Weibull distribution, we have $\overline{z} = \lambda\Gamma(1 + 1/\mu)$ and $\sigma = \lambda\sqrt{\Gamma(1 + 2/\mu) - \Gamma(1 + 1/\mu)^2}$.

We plot the optimal $LL^{best}(\overline{z},\sigma)$ as a function of $\overline{z}$ for different λ values in Fig. Suppl.3 and Fig. Suppl.4 for the log-normal and Weibull distributions, respectively. For a better comparison, we also include the data from Fig. 2 of the main text for $LL^{best}(\overline{z},\sigma)$ in the case of a Gamma distributed $w(z)$ for the smallest values of τ.

In the case of the log-normal distribution (Fig.Suppl.3), $LL^{best}(\overline{z},\sigma)$ as a function of $\overline{z}$ is very similar to the one obtained for the Gamma distribution. Specifically, we find that the maximum value of $LL^{best}(\overline{z},\sigma)$ corresponds to the same values of $\overline{z}$ and σ for both the Gamma and log-normal distributions.

For the Weibull distribution (Fig. Suppl.4), the comparison is less direct since the average value $\overline{z}$ is non-monotonic with μ for fixed λ. Nevertheless, we still find that $LL^{best}(\overline{z},\sigma)$ provides the same value of $\overline{z}$ obtained for the Gamma and log-normal distributed $w(z)$.

Based on these results, we can conclude that there is no significant difference between a Gamma, log-normal, or Weibull distributed $w(z)$. All of them lead to very similar optimal values for $\overline{z}$ and σ.

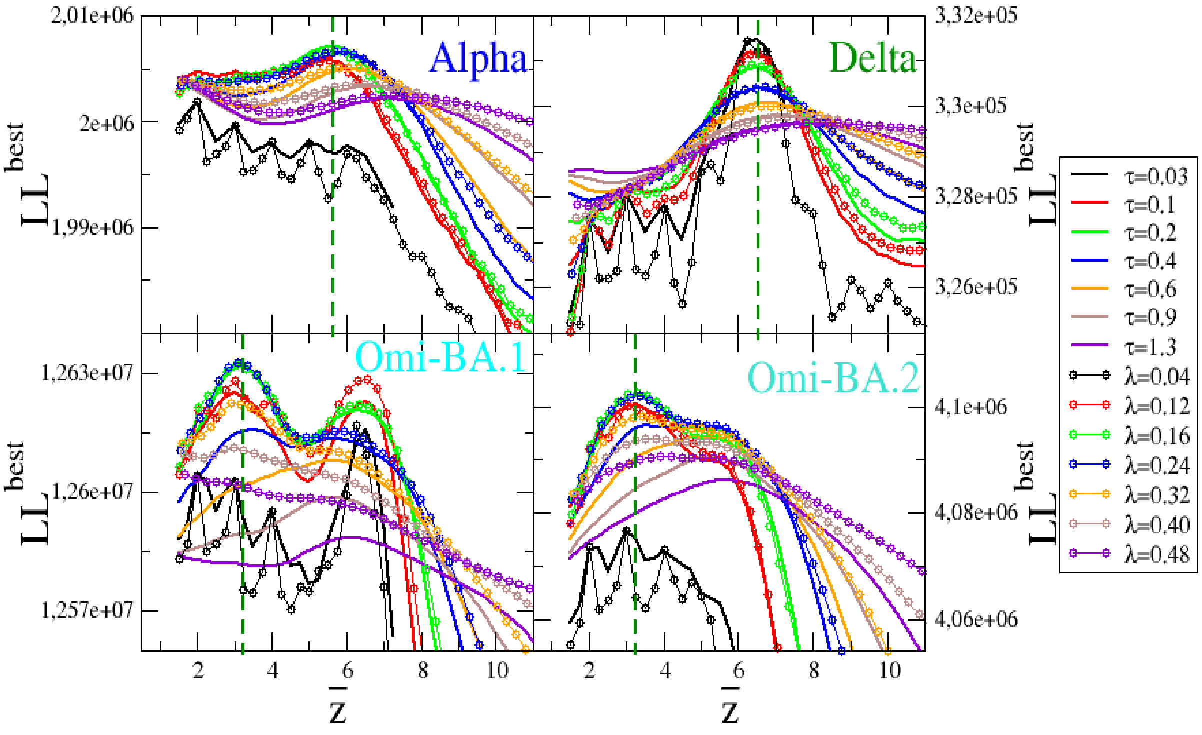

FIG. Suppl.3: (Panel a) The log-likelihood $LL^{best}(\overline{z},\sigma)$ for the optimal series $\{R_c\}$ and $\{\mu\}$, for a log-normal distributed $w(z)$ (circles) and a Gamma distributed $w(z)$ (lines). Different curves correspond to different values of λ in the case of the log-normal distributed $w(z)$ and to different values of τ in the case of the Gamma distributed $w(z)$, as reported in the legend.

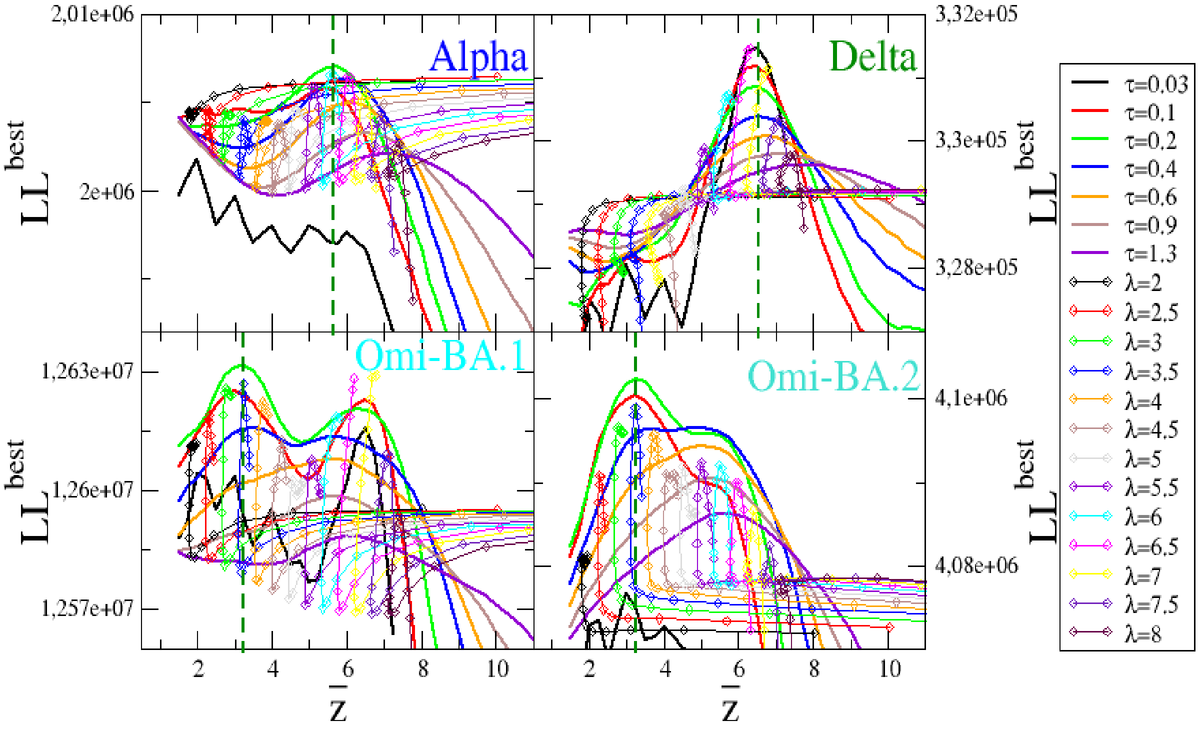

FIG. Suppl.4: (Panel a) The log-likelihood $LL^{best}(\bar{z}, \sigma)$ for the optimal series $\{R_c\}$ and $\{\mu\}$, for a Weibull distributed $w(z)$ (circles) and a Gamma distributed $w(z)$ (lines). Different curves correspond to different values of λ in the case of the Weibull distributed $w(z)$ and to different values of τ in the case of the Gamma distributed $w(z)$, as reported in the legend.

THE INFLUENCE OF σ ON $R_c(m)$

In Fig. Suppl.5, we plot the temporal evolution of the case reproduction number $R_c(m)$ during the Alpha time window. More precisely, $R_c(m)$ is the profile of $R_c(m)$ which maximizes the log-likelihood in the case that the generation time distribution is a Gamma function with $\overline{z} = 5.8$ days. Different curves in Fig. Suppl.5 correspond to different values of $\sigma \in [0.4, 3.4]$ days.

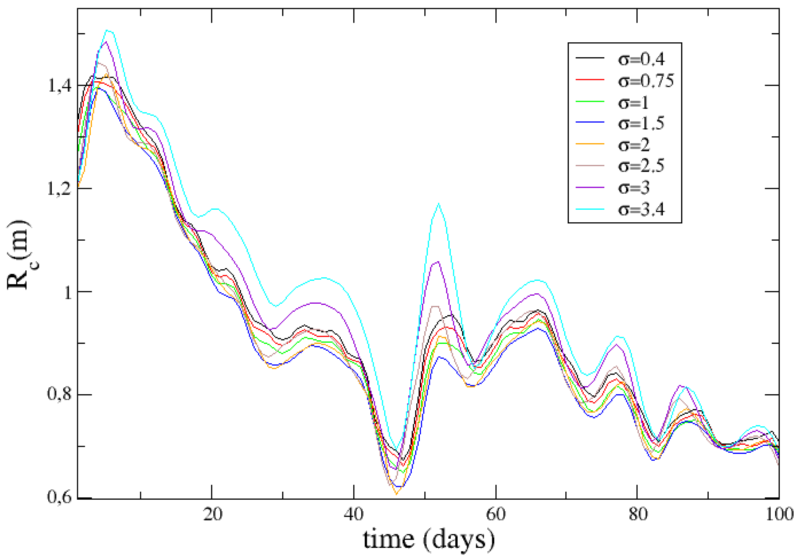

FIG. Suppl.5: The temporal evolution of $R_c(m)$ during the Alpha window in the hypothesis of Gamma distributed generation times. We assume $\overline{z} = 5.8$ days and consider different values of σ (see legend).

THE INFLUENCE OF UNREPORTED INFECTED ON $\overline{z}$

The daily incidence rate is clearly correlated to the number of performed tests. Indeed, the higher the number of performed tests, the larger the probability to identify an asymptomatic infected individual. In [1], we have developed a procedure to disentangle the daily number of infected I from the daily test number n^T. It is based on the assumption that the number of identified infected individuals during the m-th day can be viewed as the sum of two contributions $I(m) = I^{(\phi)}(m) + I^{ran}(m)$. Here, $I^{ran}(m)$ represents asymptomatic individuals who are identified as infected on the m-th day, substantially by chance, according to a random search within a population N_P.

Indicating with $I^{TOT}(m)$ the total number of new infected individuals during the m-th day, and taking into account that the search is not fully random but it is usually focused on a subset $N_P\phi_1$ of the total population, we have $I^{ran}(m) = \frac{n^T(m) I^{TOT}(m)}{\phi_1 N_P}$, with $\phi_1 < 1$. The quantity $I^{(\phi)}(m)$, on the other hand, includes all infected individuals with symptoms and all individuals who have been in strict contact with them. It is reasonable to assume that these individuals are always tested, and therefore their identified infection is not related to the daily number of performed tests. We define it as the "disentangled" incidence rate since we expect that its value does not depend on $n_T(m)$. Assuming that $I^{(\phi)}(m)$ is a fixed fraction $\phi_2 < 1$ of the total number of infected individuals, $I^{(\phi)}(m) = \phi_2 I^{TOT}(m)$, we obtain $I(m) = I^{TOT}(m)\phi_2 + I^{TOT}(m)\frac{n^T(m)}{\phi_1 N_P}$, and therefore the disentangled incidence daily rate $I^{(\phi)}(m)$ can be written as

$$I^{(\phi)}(m) = \frac{I(m)}{1 + \frac{n^T(m)}{\phi N_P}} \tag{1}$$

where $\phi = \phi_1\phi_2$ is a parameter. The value of ϕ can be fixed by imposing that $\{I^{(\phi)}\}$ is not causally related to $\{n^T\}$. This procedure leads [1] to $\phi = 5 \times 10^{-4}$, which is the value used in our analysis. More precisely, we apply our algorithm for the evaluation of the log-likelihood, considering, instead of the reported incidence rate $I(m)$, the disentangled one $I^{(\phi)}(m)$ defined in Eq.(1), and we repeat the same analysis as in Fig.2 of the main text.

Results (Fig.Suppl.6) substantially provide the same result for $\overline{z}$ as those of Fig.2 of the main text, in each of the four temporal windows. This indicates that our findings are quite stable with respect to the number of asymptomatic undetected people.

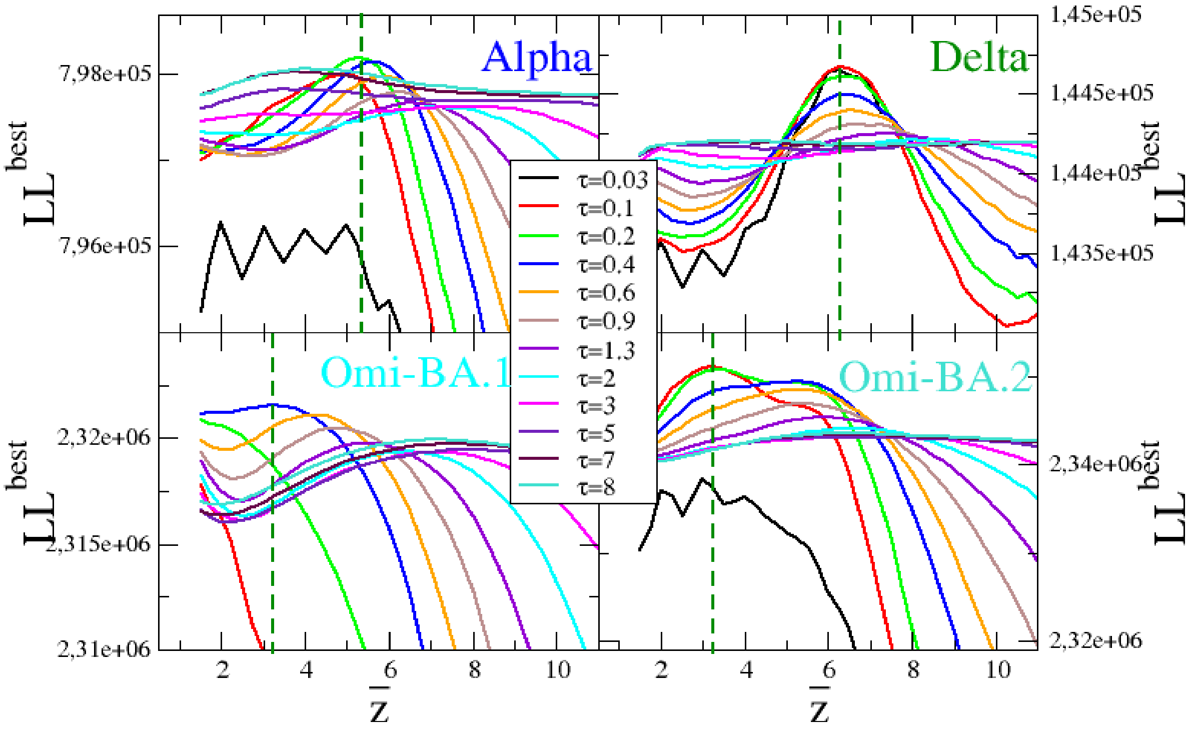

FIG. Suppl.6: The log-likelihood $LL^{best}(\overline{z}, \sigma)$, evaluated for the temporal profile of $R_c(m)$ which maximizes the likelihood for the disentangled incidence rate in Lombardy, is plotted as a function of $\overline{z} = a\tau$. The four different panels correspond to the four temporal windows: Alpha (upper left panel), Delta (upper right panel), Omicron-BA.1 (lower left panel), and Omicron BA-2 (lower right panel). Different curves in each panel correspond to different values of τ, which implies a different $\sigma = a\sqrt{\tau}$. The dashed green vertical line identifies the value of $\overline{z}$ that provides the maximum value of the log-likelihood in each panel.

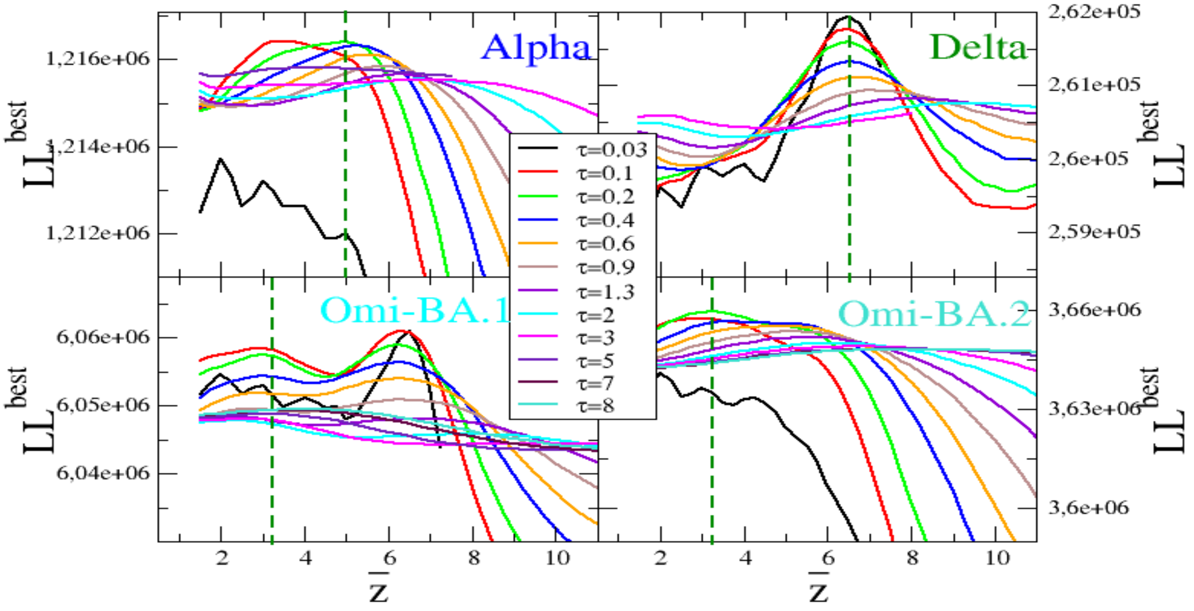

FIG. Suppl.7: The log-likelihood $LL^{best}(\overline{z}, \sigma)$, evaluated for the temporal profile of $R_c(m)$ which maximizes the likelihood for the daily incidence of SARS-CoV-2 in Campania, is plotted as a function of $\overline{z} = a\tau$. The four different panels correspond to the four temporal windows Alpha (upper left panel), Delta (upper right panel), Omicron-BA.1 (lower left panel) and Omicron BA-2 (lower right panel). Different curves, in each panel, correspond to different values of τ, which implies a different $\sigma = a\sqrt{\tau}$. The dashed green vertical line identifies the value of $\overline{z}$ which provides the maximum value of the log-likelihood, in each panel.

ANALYSIS FOR OTHER ITALIAN REGIONS

In this section, we present the same analysis performed in the main text for the region Lombardy for five other regions with the largest number of inhabitants, namely Lazio, Campania, Veneto, and Emilia-Romagna.

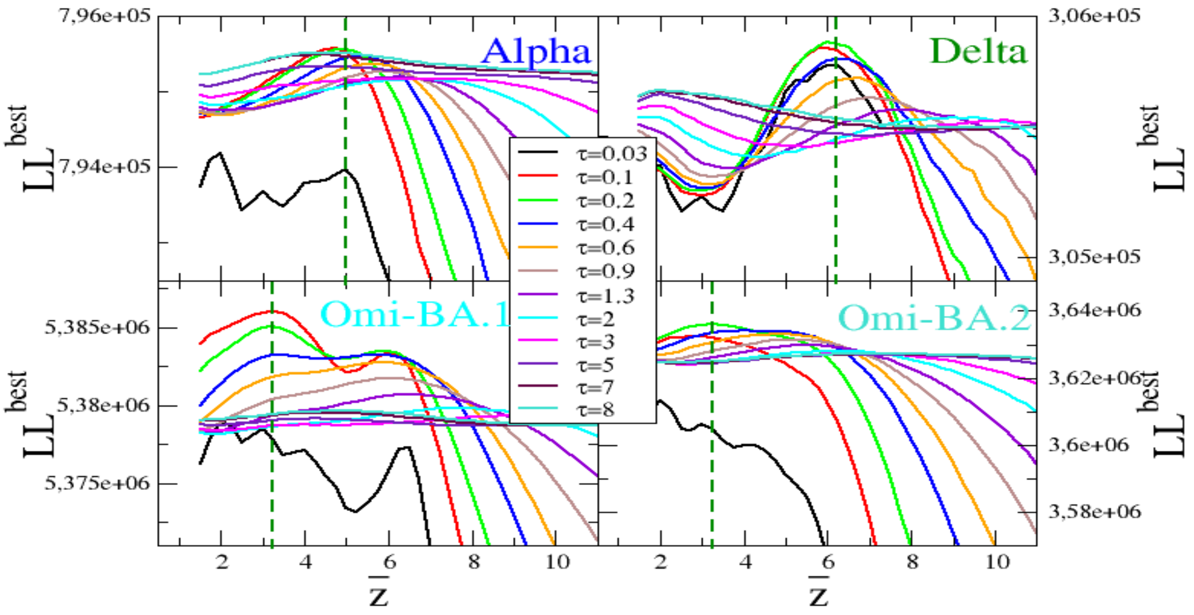

FIG. Suppl.8: The log-likelihood $LL^{best}(\overline{z}, \sigma)$, evaluated for the temporal profile of $R_c(m)$ which maximizes the likelihood for the daily incidence of SARS-CoV-2 in Lazio, is plotted as a function of $\overline{z} = a\tau$. The four different panels correspond to the four temporal windows Alpha (upper left panel), Delta (upper right panel), Omicron-BA.1 (lower left panel) and Omicron BA-2 (lower right panel). Different curves, in each panel, correspond to different values of τ, which implies a different $\sigma = a\sqrt{\tau}$. The dashed green vertical line identifies the value of $\overline{z}$ which provides the maximum value of the log-likelihood, in each panel.

[1] Lippiello, E., Petrillo, G., de Arcangelis, L.: Estimating the generation interval from the incidence rate, the optimal quarantine duration and the efficiency of fast switching periodic protocols for covid-19. Sci Rep **12**, 4623 (2022). https://doi.org/10.1038/s41598-022-08197-x

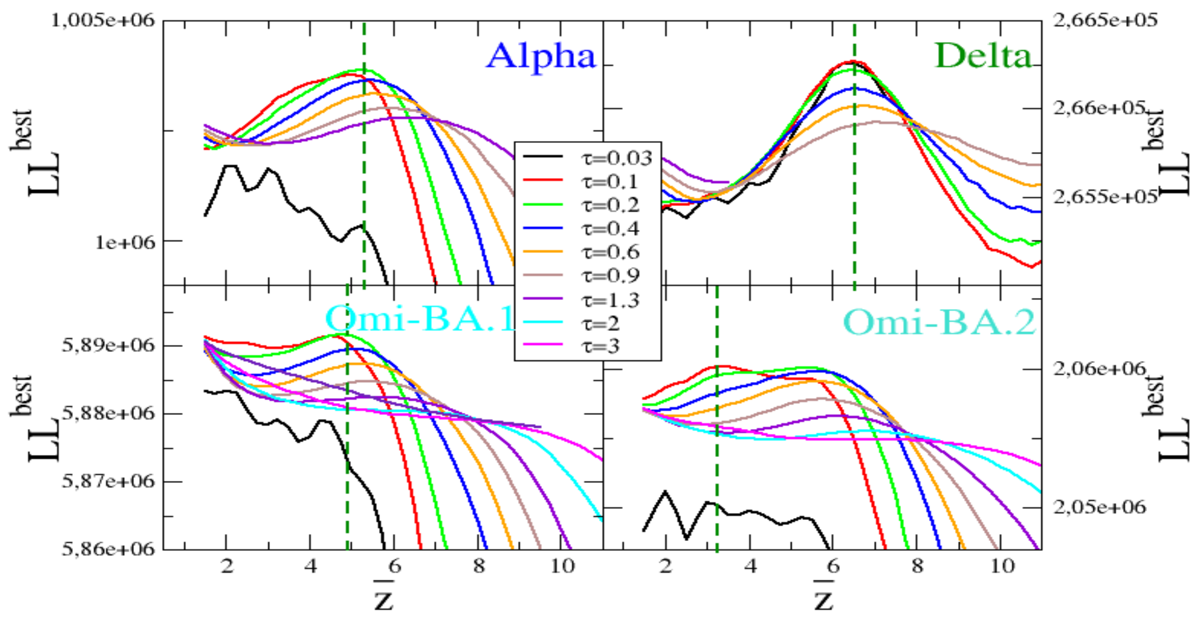

FIG. Suppl.9: The log-likelihood $LL^{best}(\overline{z}, \sigma)$, evaluated for the temporal profile of $R_c(m)$ which maximizes the likelihood for the daily incidence of SARS-CoV-2 in Emilia-Romagna, is plotted as a function of $\overline{z} = a\tau$. The four different panels correspond to the four temporal windows Alpha (upper left panel), Delta (upper right panel), Omicron-BA.1 (lower left panel) and Omicron BA-2 (lower right panel). Different curves, in each panel, correspond to different values of τ, which implies a different $\sigma = a\sqrt{\tau}$. The dashed green vertical line identifies the value of $\overline{z}$ which provides the maximum value of the log-likelihood, in each panel.

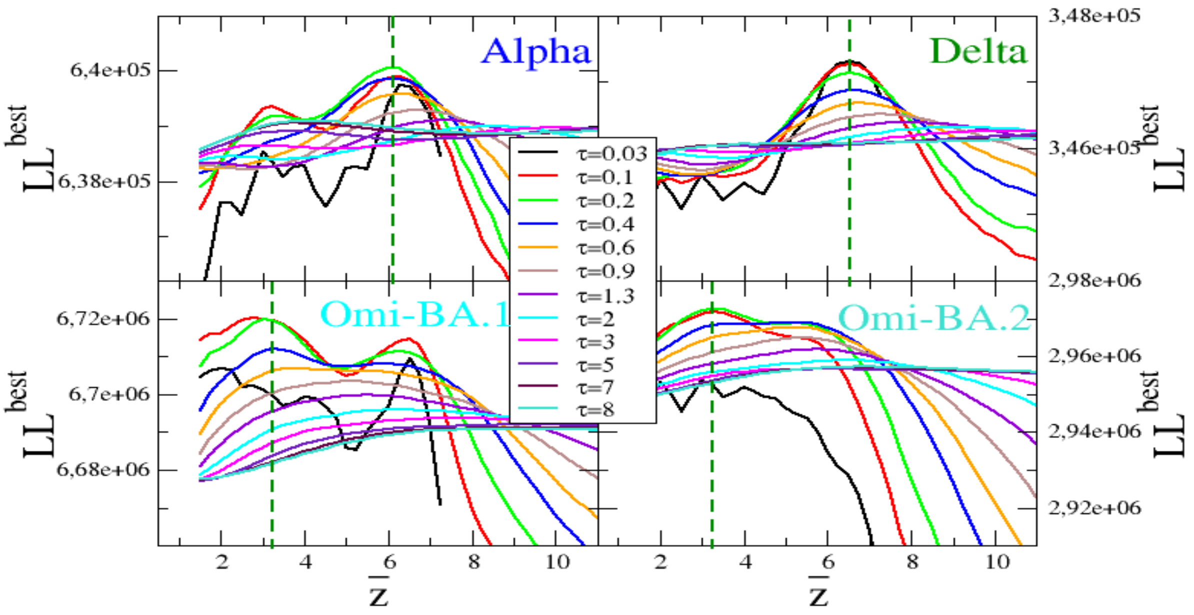

FIG. Suppl.10: The log-likelihood $LL^{best}(\bar{z}, \sigma)$, evaluated for the temporal profile of $R_c(m)$ which maximizes the likelihood for the daily incidence of SARS-CoV-2 in Campania, is plotted as a function of $\bar{z} = a\tau$. The four different panels correspond to the four temporal windows Alpha (upper left panel), Delta (upper right panel), Omicron-BA.1 (lower left panel) and Omicron BA-2 (lower right panel). Different curves, in each panel, correspond to different values of τ, which implies a different $\sigma = a\sqrt{\tau}$. The dashed green vertical line identifies the value of $\bar{z}$ which provides the maximum value of the log-likelihood, in each panel.
